# The Montreal cognitive assessment is superior to national institute of neurological disease and stroke-Canadian stroke network 5-minute protocol in predicting vascular cognitive impairment at 1 year

**DOI:** 10.1186/s12883-016-0570-y

**Published:** 2016-04-12

**Authors:** YanHong Dong, Jing Xu, Bernard Poon-Lap Chan, Raymond Chee Seong Seet, Narayanaswamy Venketasubramanian, Hock Luen Teoh, Vijay Kumar Sharma, Christopher Li-Hsian Chen

**Affiliations:** Memory Ageing and Cognition Center, Department of Pharmacology, National University Health System, Singapore, Singapore; Yong Loo Lin School of Medicine, National University of Singapore, Singapore, Singapore; Division of Neurology, National University Health System, Singapore, Singapore; Centre for Healthy Brain Ageing and Dementia Collaborative Research Centre, UNSW Medicine, The University of New South Wales, Sydney, Australia; Neuroscience Centre, Raffles Hospital, Singapore, Singapore; Department of Pharmacology, Yong Loo Lin School of Medicine, National University Health System, BLK MD3 Level 4 #04-01, 16 Medical Drive, Singapore, 117600 Singapore

**Keywords:** Vascular dementia, Acute stroke, Predictive value of tests, Cognition

## Abstract

**Background:**

The predictive ability of National Institute of Neurological Disease and Stroke-Canadian Stroke Network (NINDS-CSN) 5-minute protocol and Montreal Cognitive Assessment (MoCA) administered sub-acutely and at the convalescent phase after stroke for significant vascular cognitive impairment (VCI) at 1 year is unknown. We compared prognostic values of these tests.

**Methods:**

Patients with ischemic stroke and transient ischemic attack (TIA) received MoCA sub-acutely (within 2 weeks) and 3–6 months after stroke followed by a formal neuropsychological evaluation at 1 year. The total score of NINDS-CSN 5-minutes protocol was derived from MoCA. Moderate-severe VCI was defined as neuropsychological impairment in ≥ 3 domains. Area under the receiver operating characteristic curve (AUC) analyses were conducted to establish the optimal cutoff points and discriminatory properties of the MoCA and NINDS-CSN 5-minute protocol in detecting moderate-severe VCI.

**Results:**

Four hundre patients were recruited at baseline. Of these, 291 received a formal neuropsychological assessment 1 year after stroke. 19 % patients had moderate-severe VCI. The MoCA was superior to the NINDS-CSN 5-minute protocol [sub-acute AUCs: 0.89 vs 0.80, *p* < 0.01; 3–6 months AUCs: 0.90 vs 0.83, *p* < 0.01] in predicting for moderate-severe VCI at 1 year. At respective cutoff points, MoCA had significantly higher sensitivity than the NINDS-CSN 5-minute protocol at baseline (*p* = 0.01) and 3–6 months (*p* = 0.04).

**Conclusions:**

MoCA administered sub-acutely and 3–6 months after stroke is superior to the NINDS-CSN 5-minute protocol in predicting moderate-severe VCI at 1 year.

**Electronic supplementary material:**

The online version of this article (doi:10.1186/s12883-016-0570-y) contains supplementary material, which is available to authorized users.

## Background

Post-stroke vascular cognitive impairment (VCI) at 3–6 months after stroke is common (57.7 %) in Asian patients with mild stroke and transient ischemic attack (TIA) [1]. Approximately 25 % of stroke/TIA patients had moderate-severe VCI of sufficient severity to place them at a higher risk for cognitive decline [1, 2]. Therefore, early detection of patients with moderate-severe VCI in the subacute stroke phase (initial 2 weeks after stroke) is required for intensive reduction of vascular risk factors and improve prognosis [3].

We have previously established that the commonly used brief screening instruments, i.e., the Mini-Mental State Examination (MMSE) and Montreal Cognitive Assessment (MoCA), administered at the subacute stroke phase can predict moderate-severe VCI 3–6 months after stroke with good sensitivity, as determined by formal neuropsychological evaluations [1], which has been replicated [4]. Note that patients with the highest education level of primary school or below would award 1-point for MoCA. However, both the MoCA and MMSE take approximately 10–12 min to administer, and may not be practical for bedside screening, especially during acute stroke admission. By contrast, the National Institute of Neurological Disease and Stroke-Canadian Stroke Network (NINDS-CSN) 5-minute protocol drawn from the MoCA takes less time and has been recommended for bedside screening [5]. Two studies examined the NINDS-CSN 5-minute protocol for post-stroke VCI screening in person or over the phone and showed conflicting results [6, 7]. One study reported that NINDS-CSN 5-minute protocol performed less well than the MoCA [6], while the other reported both tests to be equivalent in detecting post-stroke VCI [7]. These inconsistent findings could be due to different scoring methods (total score of 12 and 30 respectively) and diagnosis of VCI (modified Petersen’s criteria for mild cognitive impairment and cognitive impairment defined by Clinical Dementia Rating scale respectively). Additionally, the predictive ability of NINDS-CSN 5-minute protocol for post-stroke moderate-severe VCI remains unknown. We therefore aimed to: 1) examine whether the MoCA and NINDS-CSN 5-minute protocol administered at subacute stroke phase and 3–6 months later could predict moderate-severe VCI at 1 year; and 2) compare discriminatory abilities of these two tests using their respective optimal cutoff points.

The MoCA administered at baseline has been reported to be predictive of moderate-severe VCI at 3–6 months after stroke/TIA [1]. Additionally, the NINDS-CSN 5-minute protocol was drawn from the MoCA items. Therefore, we hypothesized that the MoCA and NINDS-CSN 5-minute protocol administered at baseline and 3–6 months after stroke would be equivalent, sensitive, and predictive of moderate-severe VCI at 1 year.

## Methods

### Subjects

We recruited 400 consecutive patients (≥21 years old) with a recent ischemic stroke or TIA during the sub-acute stroke phase at the National University Hospital, Singapore. Patients were excluded if they had a major physical disability or an active psychiatric disorder that would impede cognitive testing. Eligible patients were assessed at baseline, i.e., within 2 weeks after their cerebrovascular event, 3–6 months and 12 months after stroke/TIA. At baseline their demographic and clinical outcome measures were collected along with cognitive status measured by brief cognitive test the MoCA. At 3–6 months and 12 months after stroke/TIA, their clinical outcome measures and neuropsychological performance on a formal test battery were collected. The availability of an informant was required for a patient to be included in the study.

### Standard protocol approvals and patient consent

This study was approved by the National Healthcare Group Domain-Specific Review Board (DSRB), and was conducted in conformity with the Declaration of Helsinki. Written informed consent was obtained from all participants and/or their legally acceptable representatives.

### Procedures

#### Baseline characteristics

Demographic information (age, gender, ethnicity, years of education), clinical information, vascular risk factors, and neurological status i.e. National Institute of Health Stroke Score (NIHSS) and modified Rankin Scale (mRS) were collected.

#### Clinical outcome measures

Patients were followed up at 3–6 months and 1 year after their index cerebrovascular event for their neurological status, clinical outcomes and interval events. Medical records were used to verify these events (strokes or TIA, peripheral artery disease, intracranial hemorrhage, cardiovascular risk factor, cardiac events or deaths from any of the above).

#### Cognitive measures

Patients’ cognitive status was assessed by the MoCA at baseline and 3–6 months after stroke. The MoCA has 12 items with a maximum score of 30. It consists of seven subtests: visuo-executive, naming, attention, language, abstraction, delayed recall and orientation. The MoCA was modified for Singaporean population such as replacing a test of letter fluency with semantic fluency (animals). Patients with a highest education level of primary school or below would receive an additional 1-point as education adjustment in MoCA scores. Details of MoCA modification can be found in our previous paper [8]. The NINDS-CSN 5-minute test protocol consists of three individual test items, i.e., 5-word recall (5 points), 6-item orientation (6 points), and verbal fluency [1 point if >10 words (animals) are generated in 60 s], drawn from the MoCA with a maximum score of 12 [5]. Brief description of these two tests were covered in [9]. Patients also received a formal neuropsychological battery covering seven domains locally validated for Singaporean elderly at 12 months after stroke [10], which was administered by trained research psychologists blinded to the baseline MoCA scores. The formal neuropsychological battery (Additional file 1: Table S1) has been locally validated for Singaporean and was used in stroke clinical research [1, 2, 10]. It consists of domains in executive function, attention, language, visuoconstruction, visuomotor speed, verbal and visual memory. This formal neuropsychological battery was used to define cognitive impairment. Age and education-adjusted cutoffs of 1.5 standard deviations (SD) below the established norms were used on individual tests. A result of at least half the tests failing in a domain defines domain impairment. The neuropsychological findings were interpreted by a doctoral level neuropsychologist (YD) to establish cognitive impairment. Patients with vascular cognitive impairment no dementia (VCIND) were those with objective cognitive domain impairment determined by the neuropsychological test battery without meeting the criteria for dementia. VCIND patients were further classified into VCIND mild (≤ 2 impaired domains) and VCIND moderate (≥ 3 impaired domains) [2]. VCI patients were dichotomized into no–mild VCI (no cognitive impairment (NCI) and VCIND mild) and moderate–severe VCI (VCIND moderate and dementia). Diagnoses of dementia were made according to the Diagnostic and Statistical Manual of Mental Disorders [11]. The Bayer-Activities of Daily Living Scale [12] was administered to the informant to ascertain functional disabilities primarily due to cognitive impairment.

### Statistical analyses

Data analysis was conducted using Statistical Package R version 3.2.0. Receiver operating characteristic (ROC) curve analyses were conducted to establish the optimal cutoff points and discriminatory properties of the MoCA and NINDS-CSN 5-minute protocol in detecting moderate-severe VCI. The optimal cutoff points were chosen by Youden Index [13]. Area under the operating characteristics curves (AUCs) of these tests were compared statistically [14]. Additionally, McNemar’s test was employed to compare sensitivities at the optimal cutoff points of the MoCA and NINDS-CSN 5-minute protocol.

## Results

### Subject characteristics

Recruited patients were mostly Chinese (70.3 %) and male (69.8 %), with a mean age of 59.8 ± 11.6 years and a mean of 7.7 ± 4.3 years of formal education. A majority of subjects had mild stroke (NIHSS ≤ 2, *n* = 233, 58.5 %) and minimal disability (mRS ≤ 2, *n* = 251, 63 %). Of 319 ischemic stroke patients classified using the TOAST criteria [15], most (*n* = 179, 44.8 %) patients had small artery occlusion, with a further 15.0 % (*n* = 60) with large artery atherosclerosis, 14.8 % (*n* = 59) with cardio-embolism, 5.3 % (*n* = 21) with undetermined or other determined etiology.

Two hundred ninety one out of 400 stroke/TIA patients completed a formal neuropsychological assessment at 1 year after stroke. Of these, 56 (19 %) had moderate-severe VCI (Table 1). Patients with moderate-severe VCI were older with lower education level and had higher NIHSS scores. Patients who were lost to follow-up at 1 year (*n* = 109) did not have significantly different baseline characteristics from those who completed follow-up.

**Table 1:** Sample characteristics

Characteristics	No-mild VCI (*n* = 235)		Moderate-severe VCI (*n* = 56)		Univariate Analysis *p*
Age, mean (SD)	57.46	(10.20)	68.43	(11.23)	<0.01
Gender, female	64	(27 %)	21	(38 %)	0.18
Education, *y* (mean, SD)	8.33	(4.15)	5.52	(4.02)	<0.01
Ethnicity (no., %)					0.51
Chinese	171	(73 %)	36	(64 %)	
Malay	43	(18 %)	14	(25 %)	
Indian	19	(8 %)	6	(11 %)	
Others	2	(1 %)	0	(0 %)	
Stroke classification (no., %)					0.39
SAO	112	(48 %)	23	(41 %)	
LAA	30	(13 %)	10	(18 %)	
CE	33	(14 %)	11	(20 %)	
UND	8	(3 %)	4	(7 %)	
OC	1	(0.43 %)	0	(0 %)	
TIA	51	(22 %)	8	(14 %)	
NIHSS (median)	1.00		4.00		<0.01
Baseline mRS (median)	2.00		3.00		<0.01
Mean interval (Days) between stroke/TIA and assessment (mean, SD)	2.62	(2.09)	4.02	(3.05)	<0.01
Cognitive screening test					
Baseline MoCA (mean, SD)	22.36	(4.26)	14.75	(4.13)	<0.01
Baseline NINDS-CSN 5-minute protocol (mean, SD)	9.29	(2.25)	6.41	(2.46)	<0.01
Number of cardiovascular risk factors (median)	3		3		0.14
Recurrent vascular events 3–6 months (no., %)	10	(4 %)	2	(4 %)	1.00
Recurrent vascular events 1-year (no., %)	4	(2 %)	2	(4 %)	0.54

*VCI* vascular cognitive impairment, *SD* standard deviations, *SAO* small artery occlusion, *LAA* large artery atherosclerosis, *CE* cardioembolism, *UND* undetermined etiology, *OC* other determined etiology, *TIA* transient ischemic attack, *NIHSS* National Institute of Health Stroke Score, *mRS* modified rankin scale, *MoCA* Montreal Cognitive Assessment

### Comparison of the predictive ability of the MoCA and NINDS-CSN 5-minute protocol

**Fig. 1:**
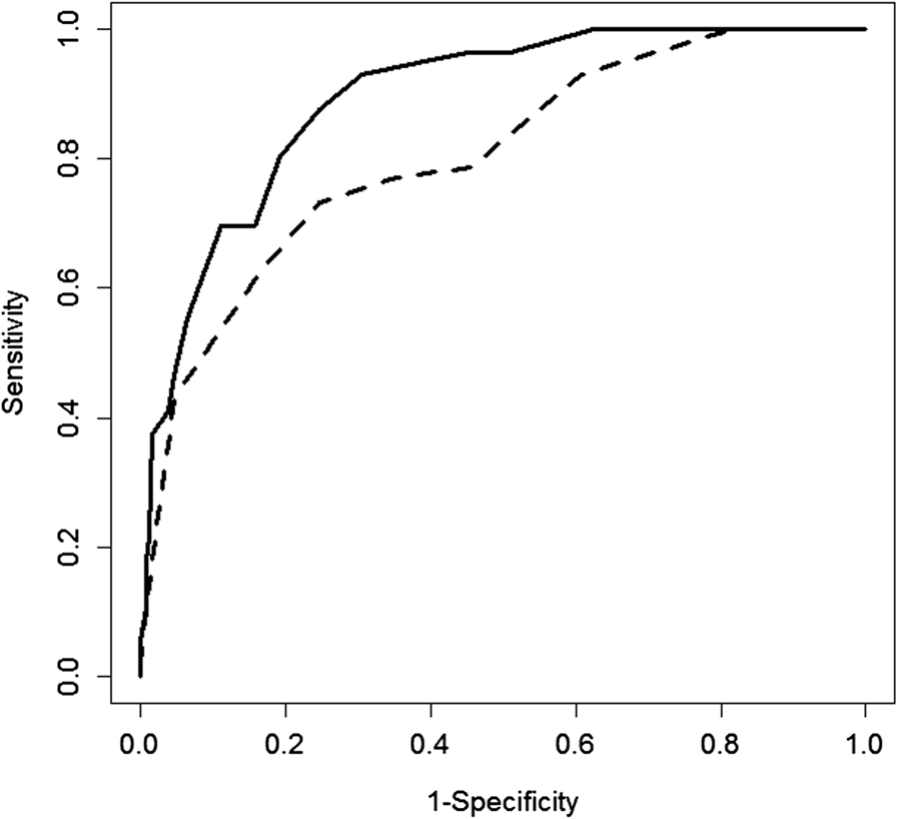
Baseline MoCA and NINDS-CSN 5-min protocol ROC curves for detecting moderate-severe VCI at 1-year. Solid line represented the ROC curve for MoCA, while the dashed line represented the ROC curve for NINDS-CSN 5-min protocol

**Fig. 2:**
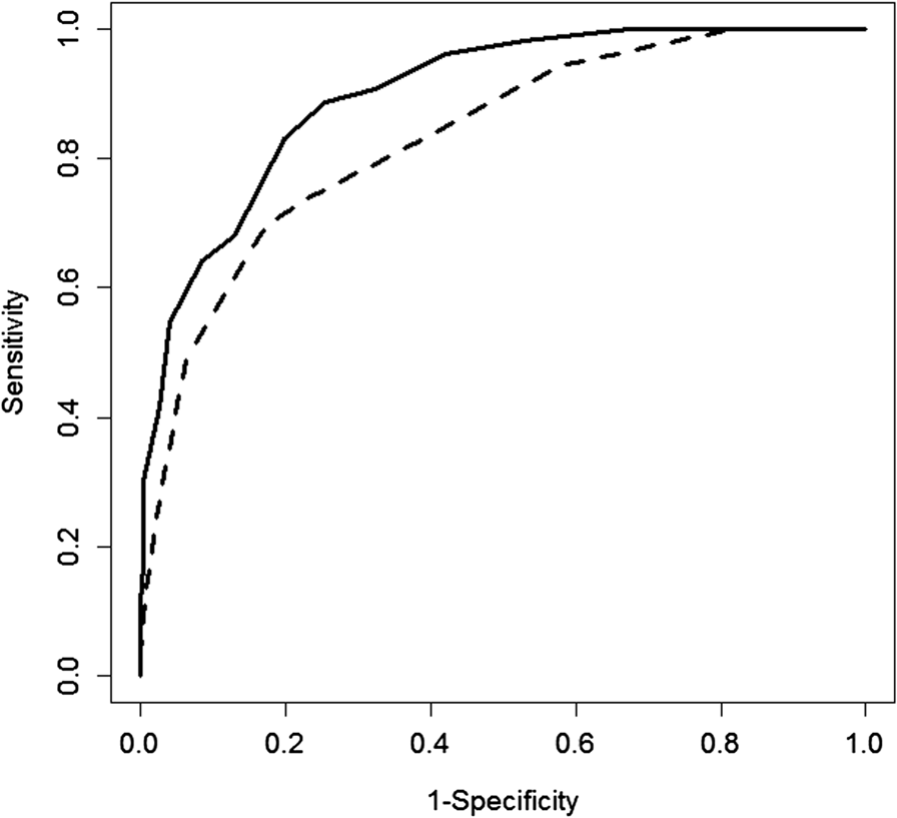
Three to six months MoCA and NINDS-CSN 5-min protocol ROC curves for detecting moderate-severe VCI at 1-year. Solid line represented the ROC curve for MoCA, while the dashed line represented the ROC curve for NINDS-CSN 5-min protocol

Figures 1 and 2 shows that the MoCA had statistically larger AUCs than the NINDS-CSN 5-minute protocol sub-acutely [AUC (95 % CI): 0.89 (0.85–0.93) vs 0.80 (0.74–0.87), *p* <0.01] and 3–6 months after stroke [AUC (95 % CI): 0.90 (0.86–0.94) vs 0.83 (0.77–0.89), *p* <0.01] for detecting patients with moderate-severe VCI at 1 year. ROC analyses repeated using age- and education-adjusted scores did not alter the results.

Table 2 shows the discriminatory properties of the MoCA and NINDS-CSN 5-minute protocol sub-acutely and at 3–6 months for predicting moderate-severe VCI at 1 year after stroke. At respective optimal cut-off points at baseline, the MoCA (<20) was superior to NINDS-CSN 5-minute protocol (<8) (*p* < 0.01). McNemar’s tests showed that the sensitivities of MoCA (<20) was significantly higher than NINDS-CSN 5-minute protocol (<8) (*p* = 0.01). At the respective optimal cutoff points at 3–6 months after stroke, the MoCA (<21) was equivalent to NINDS-CSN 5-minute protocol (<8) (*p* = 0.07). Additionally, the MoCA (<21) had significantly higher sensitivity than NINDS-CSN 5-minute protocol (<8) (*p* = 0.04). Moreover, we excluded participants who experienced at recurrent vascular event between baseline and 1 year follow up, and repeated the ROC analysis, however, results remained the same.

**Table 2:** Discriminant indices of MoCA and NINDS-CSN 5-minute protocol in detectingmoderate-severe VCI at 1-year

MoCA						NINDS-CSN 5-minute protocol					
CUT-OFF	SEN	SPEC	PPV	NPV	ACC	CUT-OFF	SEN	SPEC	PPV	NPV	ACC
(A) Baseline											
21/22	0.95	0.62	0.37	0.98	0.68	9/10	0.79	0.54	0.29	0.91	0.59
20/21	0.93	0.69	0.42	0.98	0.74	8/9	0.77	0.66	0.35	0.92	0.68
19/20^a^	0.88	0.75	0.46	0.96	0.78	7/8^a^	0.73	0.75	0.41	0.92	0.75
18/19	0.80	0.81	0.50	0.95	0.81	6/7	0.63	0.83	0.47	0.90	0.79
17/18	0.70	0.84	0.51	0.92	0.81	5/6	0.43	0.95	0.69	0.88	0.85
(B) 3–6 months											
22/23	0.91	0.68	0.40	0.97	0.72	9/10	0.81	0.64	0.35	0.93	0.67
21/22	0.89	0.74	0.45	0.97	0.77	8/9	0.74	0.77	0.43	0.92	0.76
20/21^a^	0.83	0.80	0.50	0.95	0.81	7/8^a^	0.70	0.83	0.49	0.92	0.80
19/20	0.68	0.87	0.55	0.92	0.83	6/7	0.49	0.94	0.65	0.89	0.85
18/19	0.64	0.91	0.64	0.91	0.86	5/6	0.25	0.98	0.72	0.84	0.84

^a^Optimal cut-off score

*MoCA* Montreal Cognitive Assessment, *NINDS-CSN* National Institute of Neurological Disease and Stroke-Canadian Stroke Network, *SEN* sensitivity, *SPEC* specificity, *PPV* positive predicted value, *NPV* negative predicted value, *ACC* correctly classified rate

## Discussion

The principal finding of this study is that MoCA administered in the subacute stroke phase and 3–6 months later, is more sensitive and superior to the NINDS-CSN 5-minute protocol in predicting moderate-severe VCI at 1 year, as determined by formal neuropsychological evaluations.

Our finding, that the MoCA administered at baseline is predictive of moderate-severe VCI at 1 year, is consistent with previous studies [1, 4]. Our finding that the MoCA at baseline and 3–6 months after stroke are more sensitive and superior to NINDS-CSN 5-minute protocol in detecting moderate-severe VCI at 1 year later is consistent with a previous study [6], although that study did not compare the AUCs of MoCA to NINDS-CSN using inferential statistics. Our finding differs from a recent study [7] due to different scoring method (the initial scoring method of 12 points in the present study vs. 30 points in the previous study). Additionally, the NINDS-CSN 5-minute protocol at its optimal cutoff point (<8) at baseline or 3-6 months had similar sensitivity to the findings reported by Bocti et al [16] (0.73, 0.70, and 0.72 respectively) [16]. However the specificity of the NINDS-CSN 5-minute protocol in the present study is somewhat lower than the previous findings by Bocti et al (0.75, 0.83, and 0.91 respectively). This could be due to different gold standard criteria to define cognitive impairment, i.e., a formal neuropsychological evaluation was used in the present study while MoCA < 26 was used to define cognitive impairment by Bocti and colleagues. The use of MoCA cutoff points to define cognitive impairment may have inflated the value of specificity. In view of a higher sensitivity and negative predictive value (NPV) of the MoCA relative to NINDS-CSN 5-minute test at optimal cutoff points (baseline sensitivity: 0.88 vs 0.73, NPV: 0.96 vs 0.92; 3–6 months sensitivity 0.83 vs 0.70, NPV: 0.95 vs 0.92), MoCA administered at baseline and 3–6 months after stroke would be better than the NINDSCSN 5-minute protocol in predicting for moderate-severe VCI at 1 year.

One limitation of the present study is that our sample consisted of mild stroke/TIA patients, therefore results may not be generalizable to all stroke populations. Nevertheless, approximately 1/5 of this milder stroke group had moderate-severe VCI at 1 year after stroke who may escape clinical attention without early screening at baseline or 3–6 months after stroke. Additionally, the NINDS-CSN 5-minute test protocol was developed based on expert opinion without empirical evidence [5]. The MoCA test items sensitive to cognitive pattern of “subcortical deficits” (bradyphrenia, frontal-executive and visuospatial deficits), such as visuo-executive items (Trails B, Cube, Clock) and working memory item such as Serial 7 s, that may improve screening accuracy are not included in the NINDS-CSN 5-minute test. Therefore future studies should develop an abbreviated MoCA test based on empirical evidence and validate it through independent administration of this abbreviated MoCA against a gold standard neuropsychological battery for bedside screening of VCI. Furthermore, the utility of longitudinal changes of the MoCA, NINDS-CSN 5 minute test and the empirically derived short MoCA may be examine for clinical practice.

## Conclusion

In conclusion, MoCA administered sub-acutely during stroke admission and 3–6 months after stroke has significantly higher sensitivity and is superior to the NINDS-CSN 5-minute protocol in predicting moderate-severe VCI at 1 year.

## Additional file

Additional file 1: Table S1 Cognitive domains and individual tests on the formal neuropsychological battery. (DOCX 16 kb)
