# Supplementary material for: The Montreal cognitive assessment is superior to national institute of neurological disease and stroke-Canadian stroke network 5-minute protocol in predicting vascular cognitive impairment at 1 year
Source: BMC Neurol. 2016 Apr 12;16:46. doi: 10.1186/s12883-016-0570-y (PMC4828765; doi:10.1186/s12883-016-0570-y)
Supplement: Additional file 1: Table S1 — Cognitive domains and individual tests on the formal neuropsychological battery. (DOCX 16 kb) [file 12883_2016_570_MOESM1_ESM.docx]

Table S1 Cognitive domains and individual tests on the formal neuropsychological battery

| **Cognitive Domains** | **Subtests** | **References** |
| --- | --- | --- |
| Attention | Digit span forward | Wechsler, 1997 |
|  | Digit span backward | Wechsler, 1997 |
|  | Visual span forward | Wechsler, 1997 |
|  | Visual span backward | Wechsler, 1997 |
|  | Auditory detection test | Lewis, *et al.*, 1979 |
| Language | Modified BNT | Mack, *et al.,* 1992 |
|  | Verbal fluency (Animal) | Isaacs, *et al.*, 1973 |
|  | Verbal fluency (Food) | Isaacs, *et al.*, 1973 |
| Verbal Memory | Word list immediate recall | Sahadevan, *et al.*, 1997 |
|  | Word list delayed recall | Sahadevan, *et al.*, 1997 |
|  | Word list recognition | Sahadevan, *et al.*, 1997 |
|  | Story immediate recall | Wechsler, 1997 |
|  | Story delayed recall | Wechsler, 1997 |
| Visual Memory | Picture immediate recall | Wechsler, 1997 |
|  | Picture delayed recall | Wechsler, 1997 |
|  | Picture recognition | Wechsler, 1997 |
|  | WMS-R immediate recall | Wechsler, 1997 |
|  | WMS-R delayed recall | Wechsler, 1997 |
|  | WMS-R recognition | Wechsler, 1997 |
| Visuoconstruction | WMS-R copy | Wechsler, 1997 |
|  | Clock drawing test | Sunderland, *et al.*, 1989 |
|  | WAIS-R block design | Wechsler, 1981 |
| Visuomotor Speed | Digit cancellation task | Diller, *et al.*, 1974 |
|  | Symbol digit modalities | Smith, 1973 |
|  | Maze task | Porteus, *et al.*, 1959 |
| Executive Function Domain | Frontal Assessment Battery | [Dubois and Litvan, 2000](#_ENREF_3) |

*Note*. BNT = Boston Naming Test; WMS-R = Wechsler Memory Scale-Revised; WAIS-R = Wechsler Adult Intelligence Scale-Revised.

## References

Diller, L., *et al.* (1974). *Studies in Cognition and Rehabilitation in Hemiplegia.* New York: University Medical Center, Rehabilitation Monograph, no. 50.

Dubois, B. and Litvan, I. (2000). The FAB: A frontal assessment battery at bedside. Neurology, 55, 1621–1626.

Isaacs, B. and Kennie, A. T. (1973). The Set test as an aid to the detection of dementia in old people. *British Journal of Psychiatry,* 123, 467–470.

Lewis, R. F. and Rennick, P. M. (1979). Manual for the Repeatable Cognitive Perceptual-Motor Battery. Clinton Township, MI: Axon.

Mack, W.J., Freed, D.M., Williams, B.W. and Henderson, V. W. (1992). Boston Naming Test: shortened versions for use in Alzheimer’s disease. *Journal of Gerontol*ogy, 47,154–158.

Porteus, S. D. (1959). *The Maze Test and Clinical Psychology.* Palo Alto, CA: Pacific Books.

Sahadevan, S., Tan, N. J., Tan, T. C. and Tan, S. (1997). Cognitive testing of elderly Chinese from selected community clubs in Singapore. *Annual Academy of Medicine Singapore*, 26, 271–277.

Smith, A. (1973). *Symbol Digit Modalities Test.* Los Angeles, CA: Western Psychological Services.

Sunderland, T., Hill, J. L., Mellow, A. M. and Lawlor, B. A. (1989). Clock drawing in Alzheimer’s disease. A novel measure of dementia severity. *Journal of American Geriatric Society,* 37, 725–729.

Wechsler, D. (1981). *Wechsler Adult Intelligence Scale-Revised.* New York, NY: Harcourt Brace Jovanovich.

Wechsler, D. (1997). Wechsler Memory Scale-Revised. San Antonio, TX: Harcourt Brace Jovanovich.
